# The life quality of people living with chronic disease in Africa: a systematic narrative synthesis

**DOI:** 10.11604/pamj.2024.49.115.42393

**Published:** 2024-12-10

**Authors:** Badre Bakzaza, Hanane Lemmih, Faouzi Errachidi, Omaima El Bouazzi, Saad Rachiq, Sidi Mohammed Raoui

**Affiliations:** 1 Functional Ecology and Environment Engineering Laboratory, Faculty of Sciences and Technology, Sidi Mohamed Ben Abdellah University (USMBA), Fes, Morocco,; 2 Higher Institute of Nursing Professions and Health Techniques, Meknes, Morocco

**Keywords:** Health-related quality of life, chronic diseases, systematic narrative synthesis, African studies

## Abstract

Nowadays, chronic diseases are more common and affect a huge number of individuals. They can have a negative impact on the quality of life in many domains, including physical well-being, mental health and social relationships. This systematic narrative synthesis aims to explore health-related quality of life in people with chronic diseases in Africa. Using appropriate keywords, inclusion and exclusion criteria, and an in-depth examination of bibliographics through Scopus databases were carried out according to the PRISMA chart. Thus, the 23 studies from Africa retained were published in English between January 1^st^, 2019, and October 14^th^, 2023 and they reported the quality of life-related to health in patients living with chronic disease. The results of this review revealed that patients living with chronic diseases suffer from a number of medical, psychological, and social factors that have a negative impact on their quality of life. This study highlighted the importance of giving patients with chronic diseases in Africa top priority when they come to health care services and it pointed out the need to bolster Africa's health systems by improving primary care and similarly by increasing the knowledge and skills of care staff to enhance health-related quality of life of these patients.

## Introduction

The World Health Organization (WHO) defines quality of life (QoL) as *“an individual´s perception of their position in life in the context of the culture and value systems in which they live and in relation to their goals, expectations, standards, and concerns”* [1]. Health-related quality of life (HRQoL) addresses health-related domains that are impacted by elements such as physical, psychological, social, and environmental aspects. HRQoL may thus be described as a person´s perceived quality of life, indicating satisfaction in areas of life that are likely to be impacted by health conditions. In other words, QoL is a complex concept that is influenced by an individual's physical and mental well-being, degree of autonomy, social relationship, personal convictions, and connection to prominent elements in the environment [2].

The word “chronic” is often used for a disease lasting over three months [3]. In the scholarly literature and professional societies, the term “chronic disease” is used to describe an extensive list of conditions. Common chronic diseases include arthritis, asthma, cancer, chronic obstructive pulmonary disease, diabetes, and viral diseases such as hepatitis C and human immunodeficiency virus/acquired immunodeficiency syndrome (HIV/AIDS) [4]. Likewise, Chronic diseases are often associated with non-communicable diseases, which are distinguished by their non-infectious origins [5]. In fact, the most common cause of death globally is chronic disease, which is becoming more common in all age groups, genders, and racial/ethnic groups. Although they are a major health concern in developed countries as well, the majority of deaths from chronic diseases occur in middle-to-low-income countries (LMICs) [6]. The number of deaths worldwide from chronic diseases increased from 27 million in 1990 to 39.5 million in 2016 [7]. Changes in demographics are happening in tandem with the worldwide chronic disease crisis. According to estimates from the WHO, chronic disease causes 38 million (63%) of the deaths that occur each year [7]. In the United States, at least two chronic diseases affect 40% of adults [8].

Nowadays, the co-occurrence of two or more chronic diseases in one person is becoming increasingly common because of the growing senior population brought by higher life expectancies [9]. The burden of chronic diseases predominantly impacts LMICs, which also cause over three-quarters of chronic disease-related mortality [4]. Because of limited access to healthcare, low health literacy, and socioeconomic inequality among LMICs, health systems are becoming more worried about patients with chronic diseases. Moreover, the increased prevalence of multimorbidity makes patients suffer from an additional burden of therapy in addition to the weight of disease symptoms. In effect, patients with many medical conditions are more likely to confront complicated drug schedules, the use of multiple drugs, low adherence, and adverse drug-related events [10].

Numerous studies conducted in developed countries have shown that HRQoL with chronic diseases is a reliable indicator of mortality and hospitalizations. Thus, a major result of effective disease management is most often a reduction in deterioration or improving QoL [11]. Consequently, enhancing the QoL of individuals suffering from chronic disease is imperative to managing the evolution of the disease and preventing complications [12].

One of the primary goals of health research continues to be assessing the QoL in people with chronic diseases. So various techniques and instruments for assessing life quality have been created with the aim of obtaining a comprehensive assessment encompassing physical, emotional, psychological, and social facets [13]. This study contributed to enriching the existing literature on HRQoL in Africans with chronic diseases by conducting a comprehensive review of several studies conducted in Africa. It included studies examining the medical, psychological and social factors that affect quality of life. In effect, by synthesizing the findings from the selected studies, this review aimed to enhance our understanding about the complex of taking care of person with chronic diseases.

The main objective of this systematic narrative synthesis is to study the quality of life among people living with chronic diseases in Africa. In terms of specific objectives, this study has three specific objectives. First, decorticating all facets of HRQoL in patients with chronic diseases in Africa. Second, determining the tools used to measure the HRQoL in patients with chronic diseases through the selected studies. Third, providing a framework for developing interventions to increase HRQoL outcomes for patients living with chronic diseases in Africa.

## Methods

**Review design:** this systematic review was carried out in accordance with the guidelines of Preferred Reporting Items for the Systematic Review and Meta-Analysis (PRISMA).

**Search strategy:** we used the Scopus online database as our study source. We limited the study search to studies published between June 2019 and October 14^th^, 2023. The study search was based on using appropriate keywords in English (“life quality” or “quality of life” or “health related quality of life”), and (“chronic disease”) and (“Africa”).

**Study eligibility:** to preserve articles for eligibility, certain inclusion and exclusion criteria were set. First, the date criteria are designed to keep all studies published between June 2019 and October 14^th^, 2023. Second, the geographical criteria are designed to preserve all research conducted in Africa. Third, the matching designed to retain articles in line with the study objectives. Fourth, the type of study criteria designed to keep original articles. Fifth, the language criteria designed to preserve articles published as a full manuscript in English.

**Figure 1:**
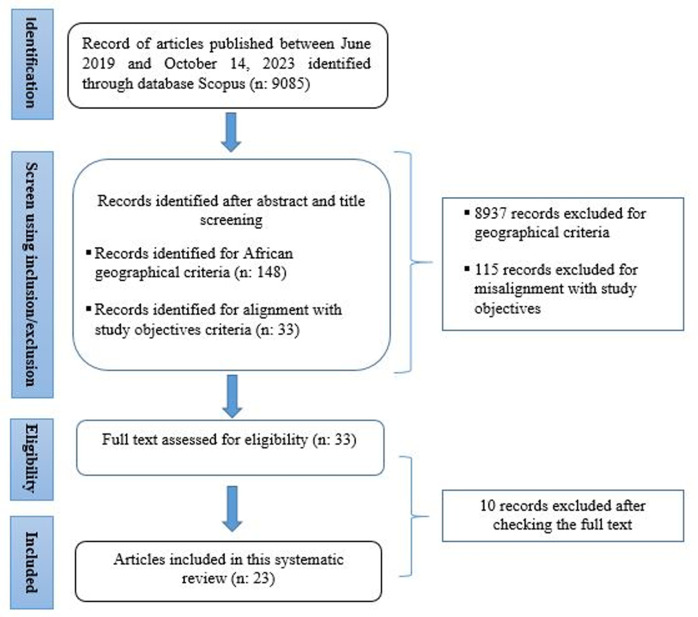
PRISMA flow diagram

**Data collection process:** the records of articles, published between June 2019 and October 14^th^, 2023, identified through Scopus were exported to the Zotero software, which allowed for the removal of redundant studies and the download of their complete texts (no duplicate results were identified). After two authors (BB and LH) independently screened the title and abstract from the collected articles to identify studies conducted in Africa and studies that align with the objective of our review. Once these two criteria have been applied, the studies that have passed the screening stage are then evaluated through a careful reading of the full manuscripts with reference to the remaining eligibility criteria. Indeed, studies that met the eligibility criteria were then included in the data extraction and synthesis. All the process of selection studies were carried out using The Preferred Reporting Items for the Systematic Review and Meta-Analysis (PRISMA) diagram, as seen in Figure 1. Ultimately, twenty-three articles n=23 were selected following a thorough screening of titles, abstracts as well as an in-depth reading.

**Synthesis of the study:** the selected articles that complied with the eligibility were extracted using some tools namely: Nvivo software and EXCEL software. The database extracted from the studies selected was based on author, year of publication, title, country of study, study type, study design, study method, sample, purpose design, findings, and study reference. Consequently, extracted data were used to complete the narrative synthesis. Thus, as qualitative research, this systematic review collects study information on the quality of life among people living with chronic diseases in Africa.

## Results

**Figure 2:**
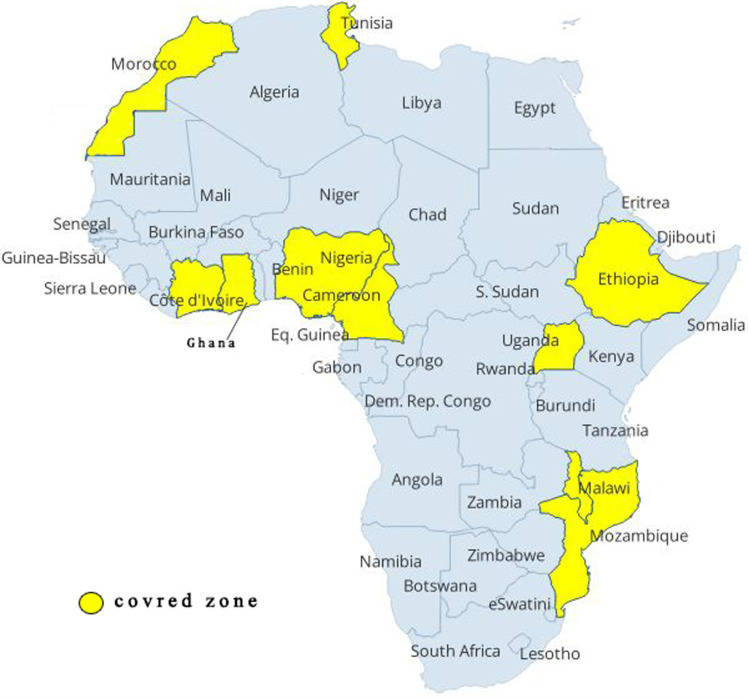
geographical distribution of African studies, carried out from January 1^st^, 2019 to October 14^th^, 2023, included in this systematic review

Despite the importance that all health systems give to the QoL, especially for people with chronic disease, few studies have drawn attention to this subject. This review led to bring out the recent studies turning around factors that can influence the QoL in patients living with chronic disease (Table 1, Table 1.1, Table 1.2, Table 1.3, Table 1.4). In general, there are 23 studies from 10 countries in Africa (Figure 2), inter alia, 10 in Ethiopia, 4 in Nigeria, and 2 in Morocco (Table 2).

**Table 1:** key findings of African studies, carried out from January 1^st^, 2019 to October 14^th^, 2023, included in the systematic review

Author and year	Setting	Population	Sample type and recruitment strategy	Study design and comparison group	Method and tools of collecting data	Findings / Results
Gebreyohannes *et al*. 2023	Ethiopia	Patients with multimorbidity (two or more chronic diseases) who visited the outpatient department and were on prescribed medicines	Simple size: 423 patients	Descriptive and analytical cross-sectional study	The Multimorbidity Treatment Burden Questionnaire (MTBQ) was used to quantify treatment burden, while the EUROQoL-5-dimensions5-Levels (EQ-5D-5L) was used to measure HRQoL	Treatment burden was inversely associated with HRQoL
Onu *et al*. 2023	Nigeria	People Living with HIV and receiving HIV-related care	Simple size: 250 patients recruited from 2 hospitals in the southeastern region of Nigeria	Descriptive and analytical cross-sectional study	Questionnaires about HRQoL, stigma, and symptoms of posttraumatic stress symptoms.	The results demonstrated a negative relationship between stigma and HRQoL. Patients reported decreased HRQoL when they endured more traumatic symptoms. The relationship between stigma and each of the HRQoL aspects was mediated by symptoms of traumatic stress
Tatsilong Pambou *et al*. 2023	Cameroon	People aged 50 and over living with HIV/AIDS	Simple size: 136 patients	A descriptive and analytical cross-sectional study in a cohort	Two questionnaires were administered to assess the level of physical activity (Ricci and Gagnon) and the brief World Health Organization Quality of Life (WHOQoL) HIV	The results make known that regular physical activity improves the QoL of people living with HIV
Pujasari and Umar, 2023	Malawi	People with HIV who met the antiretroviral therapy	Simple size: 200 patients	Descriptive and analytical cross-sectional study	Standardized scales were used to measure fatigue, pain, insomnia, antiretroviral therapy adherence, substance use, and quality of life	The QoL of HIV-positive individuals was inversely correlated with fatigue and insomnia
Ayalew *et al*. 2023	Ethiopia	Patients with pre-existing chronic non-communicable diseases	Simple size: 633 patients	A multicenter cross-sectional study	a 20-item Self-Reported Questionnaire (SRQ-20) and a 7-item Insomnia Severity Index (ISI) scale	The results demonstrate that common mental health problems and occupation, comorbidity, social support, insomnia, and quality of life are significantly correlated

**Table 1.1:** key findings of African studies, carried out from January 1^st^, 2019 to October 14^th^, 2023, included in the systematic review

Author and year	Setting	Population	Sample type and recruitment strategy	Study design and comparison group	Method and tools of collecting data	Findings/results
Ofei *et al*. 2023	Ghana	Breast cancer survivors	Simple random sampling sample size: 128	Descriptive and analytical cross-sectional study	Questionnaires on social support, religiosity, hope, optimism, benefit-seeking, Post-Traumatic Growth (PTG) and HRQoL	Perceived social support, religiosity, hope, optimism, and discovery of benefits were positively associated with PTG. Religiosity and PTG were positively associated with HRQoL
Okafor *et al*. 2023	Nigeria	People living with diabetes type 2	Simple random sampling sample size: 382	Controlled quasi-experimental study	The Audit of Diabetes-Dependent Quality of Life (ADDQoL) SF-36 scale	People with type 2 diabetes who get the educational intervention report higher HRQoL. Certain HRQoL domains showed a negative correlation with age (HRQoL declines with age). HRQoL was not significantly impacted by gender
Adamu *et al*. 2022	Ethiopia	People living with high blood pressure	Simple random sampling sample size: 376	Descriptive and analytical cross-sectional study	Questionnaire WHOQoL bref-26	The HRQoL in hypertensive patients was found to be low in all domains. The main predictors of a lower health-related quality of life of hypertensive patients were age, frequency of antihypertensive medication, limited social support, physical inactivity, co-morbidity, widowhood, khat chewing, and single status
Ahmed *et al*. 2022	Ethiopia	People with two or more chronic diseases	Simple random sampling sample size: 400	Descriptive and analytical cross-sectional study	The EQ-5D-3L scale (translation and adaptation of the English version into local Afaan Oromo and Amharic language) + consultation of patient files	Most respondents had poor overall HRQoL during the COVID-19 pandemic. Considering the influence of outbreaks on a patient's continuity of care. Indeed, during the COVID-19 outbreak HRQoL was strongly correlated with younger age, lack of formal education, shorter treatment duration, prevalence of respiratory problems, and missed medical visits during COVID-19
Chirindza *et al*. 2022	Mozambique	Children and adolescents with HIV having age from 8 to 14 years old	Convenience sampling sample size: 79 subjects of both genders aged 8-14 years	Descriptive and analytical cross-sectional study	Height, weight, mid-arm circumference (MAC) and skin folds. body mass index (BMI) Activity pedometer (SC-StepRx®, Deep River, Canada)	Compared to the baseline values of their peers without HIV, the participants who were living with HIV suffered from slow growth, low levels of habitual physical activity, and poor physical fitness, all of which negatively impacted their QoL and physiological functioning

**Table 1.2:** key findings of African studies, carried out from January 1^st^, 2019 to October 14^th^, 2023, included in the systematic review

Author and year	Setting	Population	Sample type and recruitment strategy	Study design and comparison group	Method and tools of collecting data	Findings/results
Habibu *et al*. 2022	Nigeria	Diabetic patients with and without foot ulcer	Simple size: 394 patients	Descriptive and analytical cross-sectional study	Questionnaire for chronic diseases, short form 36 (SF 36) health-related QoL questionnaire	The majority of participants, who have diabetes, report that foot ulcers negatively affect their QoL. In comparison to patients without Diabetic foot ulcer (DFU), individuals with DFU generally had lower QoL across all SF 36 dimensions, which measures the patients' physical, mental, and emotional well-being
Ayele *et al*. 2022	Ethiopia	Patients with common chronic diseases	Simple random sampling simple size 1815 patients	Descriptive and analytical cross-sectional study	Standardized WHOQoL bref Questionnaire	The environmental domain of HRQoL was most impacted by the COVID-19 pandemic. Numerous clinical and sociodemographic variables either directly or indirectly affected QoL. The physical and social relationships domains of HRQoL had an indirect positive effect on overall QoL, but the age, psychological, and environmental domains had a direct positive effect. However, the quantity of medications consumed, co-morbidity, and complications all directly lowered overall QoL. Furthermore, through the mediator factors of environment and physical health, respectively, living in a rural area and having problems both had an indirect detrimental impact on overall QoL
Bagasha *et al*. 2021	Uganda	Patients living with renal failure	Convenience sampling sample size: 364 participants (124 hemodialysis patients (HD) and 240 non hemodialysis patients non (HD))	Longitudinal cohort and comparative study	The Kidney Disease Quality of Life Short Form Ver 1.3	The QoL of Ugandan patients with End-stage renal disease (ESRD) was found to be lower in all three domains. Overall, in comparison with patients non-HD, HD patients scored lower across all three principal domains: physical health, mental health, and kidney disease. The two management groups' overall quality of life scores did not differ statistically. The only variables that were shown to be substantially correlated with QoL scores were breadwinner status, source of income, and type of hemodialysis management. These associations were noted exclusively in the primary categories of renal disease and physical health

**Table 1.3:** key findings of African studies, carried out from January 1^st^, 2019 to October 14^th^, 2023, included in the systematic review

Author and year	Setting	Population	Sample type and recruitment strategy	Study design and comparison group	Method and tools of collecting data	Findings/results
Touil *et al*. 2022	Morocco	Hemodialysis patients are diagnosed and treated for chronic renal failure	Simple size 70 patients	Descriptive and analytical cross-sectional study	Kidney Disease Quality Of Life Scale (KDQoL)	Hemodialysis patients claim that their QoL is worsening. It is influenced by several parameters including family and caregiver social support. Also, the results of the study demonstrated a correlation between life quality aspects. And age, sex, family status, level of education, the existence of a transplant plan, the presence of comorbidities, and duration of hemodialysis treatment
Aschalew *et al*. 2020	Ethiopia	People living with diabetes	Convenience sampling sample size: 408 patients	Descriptive and analytical cross-sectional study	WHOQoL-bref26 scale	The environmental and physical domains of scores were lowest compared to the social and psychological domains. Old age and living in a rural area had a significant association with a lack of QoL, while freedom from diabetes-related complications, physical exercise, general diet, and foot care were significantly associated. to a better quality of life of patients
Onyekonwu *et al*. 2020	Nigeria	People living with HIV having aged from 18 to 45 years old	Convenience sampling sample size: 389 participants	Descriptive and analytical cross-sectional study	WHOQoL-bref scale	HIV alters the QoL, particularly in the areas of physical and psychological health. The presence of comorbidities considerably reduces the QoL of these patients
Yazidi *et al*. 2020	Tunisia	People with diabetes type 1	Convenience sampling sample size: 100	Descriptive and analytical cross-sectional study	Diabetes-dependent quality of life (ADDQoL) Scale	Diabetes predictors of lack of QoL were age 33, poor socioeconomic status, longer-term diabetes, decreased daily insulin dose, human insulin treatment, higher prevalence of hospitalization for ketoacidosis, and infectious complications
Kehailou *et al*. 2020	Morroco	People with diabetes	Convenience sampling sample size: 526	Descriptive and analytical cross-sectional study	Scale ADDQoL SF-36	these factors influence negatively the QoL in diabetic person: female, advanced age, high duration of diabetes, complications and imbalance of diabetes, low level of education, physical inactivity, and professional inactivity

**Table 1.4:** key findings of African studies, carried out from January 1^st^, 2019 to October 14^th^, 2023, included in the systematic review

Author and year	Setting	Population	Sample type and recruitment strategy	Study design and comparison group	Method and tools of collecting data	Findings/results
Tusa *et al*. 2020	Ethiopia	Adults with and without diabetes	Sampling size: 359 adults with diabetes and 415 adults without diabetes	A comparative cross-sectional study	Data related to socio-demographics, behavioral, clinical factors, and HRQoL were collected through face to face interviews	The diabetic group had a significantly lower mean score in all dimensions of HRQoL than the non-diabetic group. Depression, diabetes mellitus complications, fasting blood sugar, low medication adherence, and poor diabetic self-care activity had a direct negative impact on HRQoL
Mesafint *et al*. 2020	Ethiopia	Patients with epilepsy	Convenience sampling Sampling size: 447 patients	Descriptive and analytical cross-sectional study	Interviews using the brief WHOQoL-BREF Version	The study reveals factors that influence the QoL of people with epilepsy: Perceived stigma, frequent seizures, comorbid depression and anxiety, antiepileptic drug nonadherence, antiepileptic drug side effects, and poor social support
Yao *et al*. 2019	Ivory Coast	Patients with type 2 of diabetes for at least one year who came to be diagnosed as diabetic	Convenience sampling Sample size: 168 patients	Descriptive and analytical cross-sectional study	QoL was assessed using the ADDQoL (Audit of Diabetes Dependent Quality of Life) scale	Diabetes alters most dimensions of life namely: freedom to drink and eat, financial status, physical appearance, and motivation. Regarding gender, out-of-school, and rural patients have the worst QoL. Regarding the type of medicine, insulin-treated patients have a poorer QoL versus. those treated with oral antidiabetic
Negera and Mega, 2019	Ethiopia	People living with HIV	Convenience sampling sample size: 95	Descriptive and analytical cross-sectional study	Questionnaire WHOQoL bref-HIV	Almost of the participants had poor health-related quality of life due to unemployment, comorbidity and the lack of social support from family
Gebremedhin *et al*. 2019	Ethiopia	People living with diabetes mellitus	Convenience sampling sample size: 267 patients	Descriptive and analytical cross-sectional study	WHOQoL-bref 26	All dimensions of HRQoL of diabetic patients were compromised. indeed, The study also identified important predictors such as age, disease duration, and fasting blood sugar level

**Table 2:** number of African studies, carried out from January 1^st^, 2019 to October 14^th^, 2023, included in this systematic review per country

Country	Number of studies selected in this review
Ethiopia	10
Nigeria	4
Morocco	2
Tunisia	1
Cameroon	1
Ghana	1
Ivory Coast	1
Malawi	1
Mozambique	1
Uganda	1

**Figure 3:**
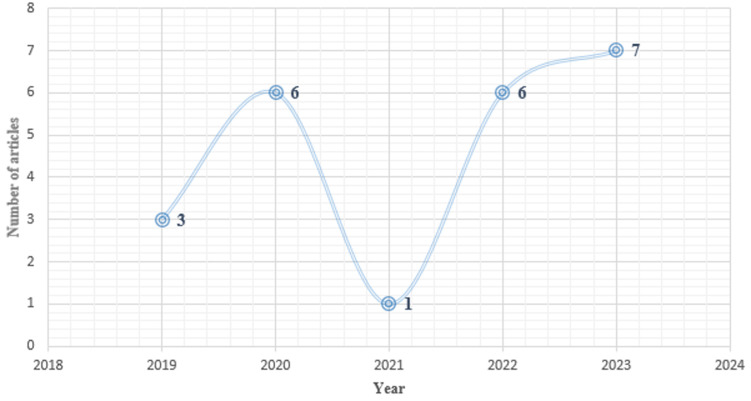
distribution per year of African studies, carried out from January 1^st^, 2019 to October 14^th^, 2023, included in this systematic review

The distribution per year of the studies selected for this review is as follows: 7 in 2023, 6 in 2022, 1 in 2021, 6 in 2020, and 3 in 2019 (Figure 3).

For the subject of articles retained in this review were: 8 studies about QoL in diabetic patients, 6 about QoL in people living with HIV, 4 studies about QoL in people with two or more chronic diseases, 2 studies about renal failure, and 1 study for each of blood pressure, epilepsy, and breast cancer.

## Discussion

**Quality of life (QoL) in diabetic patients:** every study on diabetes that was retrieved for this review showed that patients with diabetes in Africa experience daily declines in their QoL [14-21]. To measure the HRQoL in diabetic people, the majority of searchers employed the Audit of Diabetes Dependent Quality of Life scale (ADDQoL) and World Health Organization Quality of Life-Brief 26 (WHOQoL-bref 26) with the exception of one that used in-person interviews to gather information on sociodemographics, behavioral, clinical, and HRQoL factors.

This study revealed factors that influence the life quality of diabetic people in Africa such as old age, longer-term diabetes fasting blood sugar level, poor socioeconomic status, living in a rural area, human insulin treatment, higher prevalence of hospitalization for ketoacidosis and infectious complications had a significant association with lower QoL. In contrast, freedom from diabetes-related complications, physical exercise, general diet, and foot care were significantly associated with a better quality of life for patients. This synthesis's recommendations to improve the QoL for diabetic patients were emphasized, including the availability of standard treatments and the avoidance of complications from the disease. Furthermore, it is essential to provide diabetic patients with accurate lifestyle advice, paying particular attention to elderly patients and those living in rural areas.

**Quality of life (QoL) in people living with HIV/AIDS:** regarding studies that brought out the QoL in people living with HIV/AIDS in Africa, results showed that the HRQoL in these people is worse either physical or psychological [22-27]. The searchers in this review used many instruments to evaluate QoL and to correlate it with some variables. Most of the authors used the questionnaire WHOQoL bref-HIV and others combined this tool with standardized scales to evaluate fatigue, insomnia, stigma, and post-traumatic stress symptoms.

Overall, the studies included in this review found that the poor life quality of people living with HIV/AIDS is related to unemployment, comorbidity, traumatic stress, stigma, poor physical fitness, fatigue, insomnia, and the lack of social support from family. On the other hand, the study that correlated HRQoL in patients with HIV/AIDS to physical activity identified the positive impact of this variable on people living with HIV/AIDS. Additionally, the study that aim to explore the HRQoL in subjects aged 8-14 years identified slow growth as the principal factor that influence negatively the QoL in this group of patients. This result was found by combining some tools as follows: height, weight, mid-arm circumference and skin folds, body mass index activity pedometer, and HRQoL questionnaire. Therefore, younger patients may need mental health services to manage their illness.

**Quality of life (QoL) in hypertensive patients:** the searcher in the study that looked into the factors that influence HRQoL in hypertensive people used WHOQoL bref-26 questionnaire [28]. So, this study reveals that age, duration of antihypertensive treatment, low social support, physical inactivity, co-morbidity, being a widow, khat chewing, and being single were the principal predictors of lower QoL in hypertensive people in Africa. Indeed, the study suggests that, in order to improve the QoL, government, non-governmental organizations, and health professionals should give careful attention to hypertensive people, and the healthcare system should introduce a new more relevant therapeutic approach [28].

**Quality of life (QoL) in patients with renal failure:** authors in studies retrieved for this review used The Kidney Disease Quality Of Life scale (KDQoL) to determine the quality of life in people suffering from kidney disease [11,29]. The study participants conveyed that their quality of life is declining in certain areas due to various factors, including age, gender, familial relationships, educational attainment, the existence of a transplant plan, the presence of comorbidities, and the length of time they receive hemodialysis. It was discovered that there was a relationship between the overall quality of life score, the “Burdens of Kidney Disease” score, and the “Effects of Kidney Disease on Daily Living” score. There is not, however, any conclusive link with caregiver support. Therefore, the family and the medical staff must be more actively involved in the management of these patients in order to preserve their high quality of life. This management must be based on the psychological support process.

**Quality of life (QoL) in epileptic patients:** the author of the study that explores QoL in epileptic patients chose interviews using WHOQoL-BREF [30]. This study revealed that the factors which have an impact on the quality of life of epileptic patients are perceived stigmatisation, frequent crises, comorbid depression, and anxiety, non-adherence to anti-epileptic drugs, side-effects of anti-epileptic drugs, and lack of social support.

**Quality of life (QoL) in women with breast cancer:** the study that explored the QoL in women with breast cancer showed that perceived social support, religiosity, hope, optimism, and discovery of benefits were positively associated with post-traumatic growth. Likewise, religiosity and post-traumatic growth were positively associated with HRQoL [31]. To prove that, the author used questionnaires on social support, religiosity, hope, optimism, benefit-seeking, post-traumatic growth, and WHOQoL scale.

**Quality of life (QoL) in patients with two or more chronic diseases:** for studies targeting QoL in people with two or more chronic diseases, the results revealed that these individuals have low HRQoL overall [32-35]. In summary, the investigations indicated that a number of clinical and sociodemographic variables either directly or indirectly affected QoL. In fact, there is a strong correlation between comorbidity, insomnia, social support, and quality of life as well as common mental health disorders and occupation. In general, the authors in these studies used WHOQoL scale.

## Conclusion

This systematic narrative synthesis assessed the life quality of people living with chronic diseases in Africa. In fact, all studies included in this synthesis show that chronic disease affects various parameters such as physical well-being, psychological status, social relationship, and economic status in a patient´s life, thus touching the QoL. It is for these reasons that the management of these patients must include not only homeostasis, and the treatment of somatic complications, but also the evaluation of individual patient satisfaction, which results in an improvement in their quality of life. Thereby, findings of the present synthesis showed the need to investigate further the QoL of patients with chronic diseases and it is necessary to include the recommendations of the investigation of HRQoL as part of the routine care system.

### What is known about this topic

- Poor health-related quality of life (HRQL) is frequent among patients living with chronic diseases;
- Healthcare systems need to pay more attention to patients living with chronic diseases.

### What this study adds

- Studies on the quality of life of patients living with chronic diseases are rare in Africa;
- The quality of life of patients living with a chronic disease could be assessed using generic or specific instruments;
- The holistic biopsychosocial approach needs to be promoted by healthcare systems in Africa to improve the quality of life of patients living with chronic disease.
